# Carpet Tack Sign

**DOI:** 10.31138/mjr.32.3.278

**Published:** 2021-08-25

**Authors:** Joydeep Samanta, Arghya Chattopadhyay, Debashish Mishra, Dipankar De, Sanjay Jain

**Affiliations:** 1 Clinical Immunology and Rheumatology Unit, Post Graduate Institute of Medical Education and Research, Chandigarh, Punjab, India; 2 Department of Dermatology, Post Graduate Institute of Medical Education and Research, Chandigarh, Punjab, India

**Figure 1.**
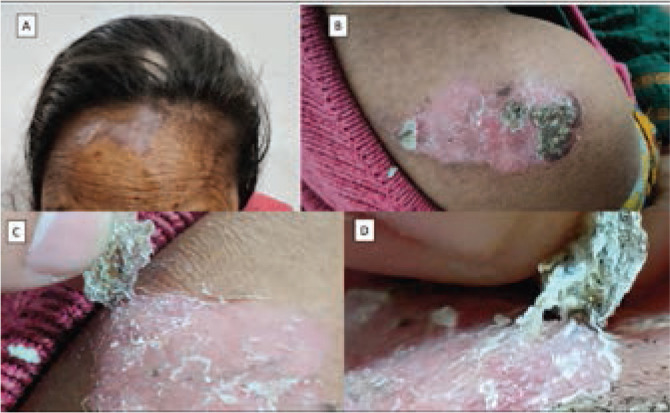
**(A)** Discoid lupus lesion over forehead. **(B)** Discoid lupus rash over shoulder. **(C,D)** On removal of the scale from the discoid lesion, horny plugs were seen occupying the hair follicles suggestive of “carpet tack sign”.

A 40-year-old-woman presented with a history of oral ulcer, scarring alopecia, photosensitive malar rash, and a non-itchy discoid shaped rash over the forehead (**Figure 1A**), right shoulder (**Figure 1B**), and anterior chest wall of three months duration. On removal of the scale from the discoid lesion, horny plugs were seen occupying the hair follicles suggestive of “carpet tack sign” (**Figure C,D**). Her investigations revealed anti-nuclear antibody (ANA) positivity. Subsequent testing for her autoantibody profile revealed positivity for anti Ro and anti-double stranded DNA. Serum complements were normal. Further investigations revealed leukopenia (Total leukocyte count 3.2 x 10^3^/μL) without any other haematological abnormality. The rest of the laboratory parameters including renal function were within the normal limit. A diagnosis of cutaneous lupus erythematosus (CLE) was made, and she was started on hydroxychloroquine (5mg/kg/day) and photo protection.

The Discoid lupus erythematosus (DLE) is the most prevalent type of chronic CLE and one of the lupus specific skin lesions as per Gilliam classification.^1^ It is 2–3 times more common than systemic lupus erythematosus (SLE). Lesions are located mainly over the face, scalp, and ears. Lesions above the neck are termed localised (about 80% of DLE cases), whereas lesions both above and below the neck are known as generalised DLE (about 20% of DLE cases). ANA positivity is found in around 10–30% of cases and underlying diagnosis of SLE is present in around 5% of cases of localised DLE and 20% of cases of systemic DLE.^2^ Common triggers for DLE are trauma (Koebner effect), UV radiation, infection, burns, exposure to extreme cold. Carpet tack sign is a characteristic sign of DLE. It is also known as “cat’s tongue sign” or “tin tack sign”.^3^ It occurs due to the accumulation of scales in the hair follicles with resultant loss of hair. Later on, when scales are peeled off, typical keratotic spikes become visible producing characteristic “carpet-tack sign”.^4^ Histologically, these lesions are characterised by parakeratosis, follicular hyperkeratosis, dense patchy perivascular, and perifollicular lymphocytic infiltrate in the dermis with dermal mucin deposition. Seborrheic dermatitis and localized pemphigus foliaceous can have similar findings, except for the bleeding on the removal of scale due to adherent nature of DLE lesions.^5^
